# Near-Field Mixing in a Coaxial Dual Swirled Injector

**DOI:** 10.1007/s10494-024-00596-6

**Published:** 2024-11-04

**Authors:** Sylvain Marragou, Thibault Frédéric Guiberti, Thierry Poinsot, Thierry Schuller

**Affiliations:** 1 Institut de Mécanique des Fluides de Toulouse, IMFT, CNRS, Université de Toulouse, Toulouse, France; 2 Clean Energy Research Platform, King Abdullah University of Science and Technology (KAUST), Thuwal, 23955-6900 Kingdom of Saudi Arabia; 3 Mechanical Engineering Program, Physical Science and Engineering Division, King Abdullah University of Science and Technology, Thuwal, Saudi Arabia; 4 Institut Universitaire de France (IUF), Paris, France

**Keywords:** Mixing, Coaxial injector, Swirled, Hydrogen, Raman

## Abstract

Improving mixing between two coaxial swirled jets is a subject of interest for the development of next generations of fuel injectors. This is particularly crucial for hydrogen injectors, where the separate introduction of fuel and oxidizer is preferred to mitigate the risk of flashback. Raman scattering is used to measure the mean compositions and to examine how mixing between fuel and air streams evolves along the axial direction in the near-field of the injector outlet. The parameters kept constant include the swirl level $S_e = 0.67$ in the annular channel, the injector dimensions, and the composition of the oxidizer stream, which is air. Experiments are carried out in cold flow conditions for different compositions of the central stream, including hydrogen and methane but also helium and argon. Three dimensionless mixing parameters are identified, the velocity ratio $u_e/u_i$ between the external stream and internal stream, the density ratio $\rho _e/\rho _i$ between the two fluids, and the inner swirl level $S_i$ in the central channel. Adding swirl to the central jet significantly enhances mixing between the two streams very close to the injector outlet. Mixing also increases with higher velocity ratios $u_e/u_i$, independently of the inner swirl. Additionally, higher density ratios $\rho _e/\rho _i$ enhance mixing between the two streams only in the case without swirl conferred to the central flow. A model is proposed for coaxial swirled jets, yielding a dimensionless mixing progress parameter that only depends on the velocity ratio $u_e/u_i$ and geometrical features of the swirling flow that can be determined by examining the structure of the velocity field. Comparing the model with experiments, it is shown to perform effectively across the entire range of velocity ratios $0.6 \le u_e/u_i \le 3.8$, density ratios $0.7 \le \rho _e/\rho _i \le 14.4$, and inner swirl levels $0.0 \le S_i \le 0.9$. This law may be used to facilitate the design of coaxial swirled injectors.

## Introduction

**Fig. 1:**
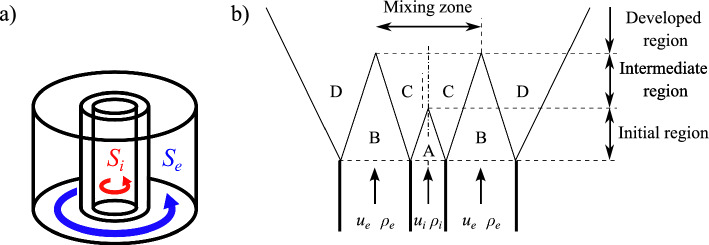
(**a**) Schematic of the dual swirl annular flow with co-rotating annular and central jets. (**b**) Structure of the flow in non-swirling coaxial jets. A: Inner potential core, B: Outer potential core, C: Inner mixing layer, and D: Outer mixing layer

Swirling jets are used in many industrial systems such as turbomachineries, mixing tanks, pumps, noise dampers, and cooling systems. The swirl motion conferred to the annular stream of a coaxial injector (Fig. 1a) enhances mixing between the annular and central jets because it increases the entrainment velocity (Park and Shin 1993), turbulence at small scales (Ribeiro and Whitelaw 1980), and the large scale vortical structures that develop in shear layers, such as precessing vortex cores (Fröhlich et al 2008). However, the impact of swirl of the central injection on mixing remains inadequately documented.

Dual swirl coaxial injector configurations (Fig. 1a) where both streams of a coaxial injector are swirled, have been utilized in combustion systems for many years in gas turbines, rocket engines, and industrial burners (Degeneve et al 2019; Chen et al 2019; Yang et al 2020; Xue et al 2022; Lin et al 2022; Leroy et al 2022). This configuration has recently received increasing attention, particularly for hydrogen-fueled burners, where separate injection H$_2$ and oxidizer is favored to mitigate the risks of flashback (Marragou et al 2022; Leroy et al 2022), an issue of paramount concern in the development of hydrogen powered burners (Tuncer et al 2009; Eichler et al 2012). However, downstream of the coaxial injector, rapid mixing between the two jets is imperative to achieve a quasi-homogeneous mixture prior to combustion, which is crucial for maintaining local gas temperatures and NO$_{x}$ emissions low (Chiesa et al 2005; Tuncer et al 2009; Magnes et al 2023). In this context, a low NO$_{x}$ injector where both H$_2$ and air streams are swirled, has been patented by IMFT and Safran Helicopter Engine (Richard et al 2021, 2023), and extensively studied in the last years (Marragou et al 2022, 2023a, b; Aniello et al 2023; Magnes et al 2023). In order to achieve low levels of NO$_{x}$ emissions, the mixing between the two swirled jets is critical, and the mixing mechanisms at play must be comprehensively understood.

Due to its technological significance, the mixing between two coaxial non-swirling (Fig. 1b: $S_{i}$ = $S_{e}$ = 0) jets has been extensively studied. Several studies have identified the square root of the momentum ratio $J^{1/2} = \left( \rho _i/\rho _e \right) ^{1/2} u_e/u_i$ as the primary parameter driving the mixing between coaxial jets (Hill 1972; Rehab et al 1997; Villermaux and Rehab 2000; Schumaker and Driscoll 2012). In the absence of swirl, the mixing process of coaxial jets can generally be divided into three zones (Azim 2019), namely the initial, intermediate, and developed regions as schematically shown in Fig. 1b. The initial region begins at the outlet of the injector and ends with the disappearance of the inner potential core (zone A in Fig 1b). The intermediate region begins downstream of the initial region and ends with the disappearance of the outer potential core (zone B in Fig 1b) and the beginning of the developed region. This flow structure is however not realized in practice in the case of swirled jets due to the absence of top-hat velocity profiles and the very quick disappearance of pure gas A and B zones when swirl is confered to these streams. For non-swirling co-axial jets, Alpinieri (1964) has shown that the mixing rate increases with (i) The velocity ratio $r_u = u_e/u_i$, where $u_e$ is the bulk velocity in the external channel and $u_i$ is the bulk velocity in the inner channel, and with (ii) The density ratio $r_\rho = \rho _e/\rho _i$, with $\rho _e$ the gas density in the external channel and $\rho _i$ the gas density in the inner channel. Without swirl, Crow and Champagne (1971) emphasize that the mass exchange mechanism between the annular and the central jets (zones A and B in Fig 1b) is driven by large coherent structures that develop in the shear layer separating the inner and the annular jets (zone C in Fig. 1b). Villermaux and Rehab (2000) confirmed that mixing is due to the dilution of the inner jet by the fluid from the annular jet. Recently, Chouaieb et al (2017) conducted a numerical investigation that focuses on the influence of the swirler position and its geometry on the mixing of a coaxial dual-swirl injector. They demonstrated that, for a constant momentum ratio between the central and annular injection channels, mixing occurs more rapidly with lighter gases injected through the central channel.

A near-field approach can be used to model the mixing of coaxial jets and also predict the stoichiometric line disappearance distance, also called stoichiometric length $L_s$ (Villermaux and Rehab 2000; Schumaker and Driscoll 2009). The resulting scaling law for the dimensionless stoichiometric length is $L_s/d_i \propto \left( J^{1/2} X_s \right) ^{-1}$, where $d_i$ is the diameter of the central injection tube and $X_s$ the stoichiometric mole fraction. Recently, this approach has been adapted and validated for a dual swirl coaxial injector similar to the HYLON injector and developed for methane-oxycombustion (Degenève et al 2019).

The near-field approach used by Villermaux and Rehab (2000) is adapted herein and applied to the case of dual swirl coaxial jets for the scaling of a mixing progress variable *C* defined by:

1 $$\begin{aligned} C \left( y \right) = \displaystyle {\frac{1 - \textrm{max} \left( X_{i} \right) }{1 - X_{i}^{\infty }}} \end{aligned}$$

where max$\left( X_{i} \right)$ denotes the maximum value of the molar fraction $X_i$ profile at a given height $y/d_i$ above the coaxial injector outlet and *X*_*i*_^*∞*^ is the molar fraction of the perfectly mixed jets. The subscript *i* refers to the species injected through the inner channel. The mixing progress *C* can be obtained by measuring the concentration along the jet axis. This is done here using a Raman scattering technique.

The objective of this study is to develop a physics based model for the mixing of swirling coaxial jets with fuel injected in the center. Experiments are conducted for a range of inner swirl levels $S_i$, velocity ratios $r_u = u_e/u_i$, and density ratios $r_\rho = \rho _e/\rho _i$ when the composition of the central jet is varied. The experimental setup is presented in Sect. 2. A parametric study is carried out in Sect. 3 in order to identify the driving parameters for the mixing progress. The mixing progress variable based on *C* is analyzed in Sect. 4 for different flow operating conditions. Finally a model is proposed in Sect. 5 predictions are compared with experiments.

## Experimental Setup

### Coaxial Dual Swirl HYLON Injector

**Fig. 2:**
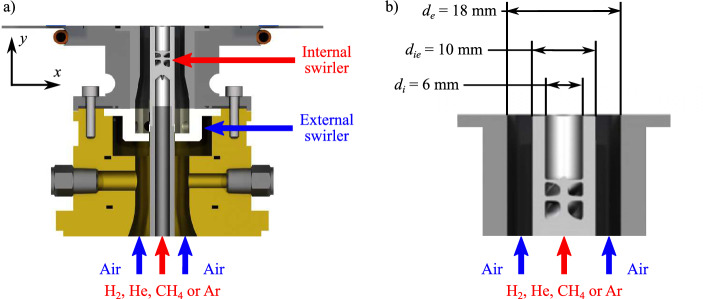
Coaxial dual swirl HYLON injector (**a**) with main dimensions (**b**)

Experiments are carried out with the experimental test bench MIRADAS (Oztarlik et al 2020; Schuller et al 2022) installed at IMFT and equipped with the HYLON injector described by Marragou et al (2022, 2023a) shown in Fig. 2. In this coaxial injector, the two streams are swirled to stabilize H$_2$-enriched methane flames (Marragou et al 2022) and full H$_2$ flames (Marragou et al 2023a, b; Aniello et al 2023; Magnes et al 2023). In this work, the injector is only operated in cold flow conditions to study the mixing dynamics between the central jet flow and annular coaxial air. The central channel can be supplied with methane, hydrogen, helium, or argon to vary the density ratio between the central and annular jets.

Swirl is imparted to the flow injected inside the chamber by a radial vane installed inside the annular channel and by an axial swirl vane inside the central tube. The external swirl level is fixed and estimated to $S_e = 0.67$ at the outlet of the annular channel from geometrical considerations. The central swirl level is varied from $S_i = 0.0$ to $S_i = 0.9$. The swirl motions of both inner and outer flows rotate in the same counterclockwise direction (co-swirls) as illustrated in Fig. 1a. Both outer and inner swirlers geometries and details on the estimations of the swirl numbers are provided in Marragou (2023). These estimates rely on a linear velocity profile for the azimuthal velocity and a uniform top hat profile for the axial velocity. Effects of the density, turbulent fluctuations and pressure are neglected in these estimations (Vignat et al 2022) The main dimensions of the injector are indicated in Fig. 2b. The inner diameter of the central tube is $d_i = 6$ mm and the outer diameter of the central tube is $d_{ie} = 10$ mm. The diameter of the annular injection channel is fixed to $d_e = 18$ mm. The outlet of the central tube is located at the same axial position as the outlet of the annular air channel as in Marragou et al (2023b), corresponding to the axial position of the bottom of the combustion chamber. The swirled central and annular streams mix in a square combustion chamber of 78-mm inner width and 150-mm length. The chamber is equipped with two fused silica windows to provide optical access, and two black windows with small slits for the passage of a laser beam. A convergent section is installed at the outlet of the chamber with a contraction ratio of the cross section area equal to 0.69.

### 1D1S Raman Scattering System

**Fig. 3:**
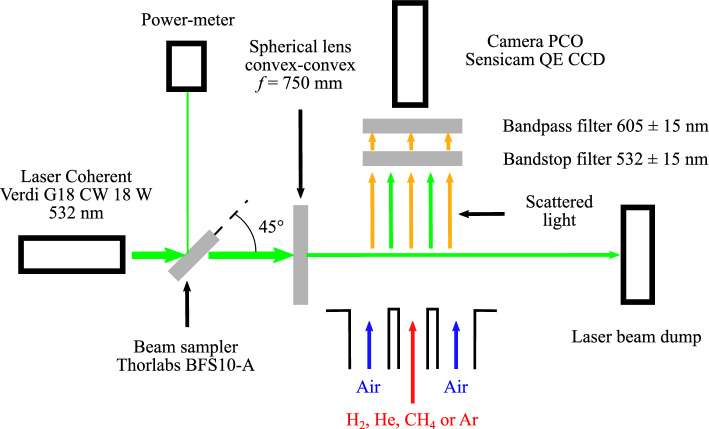
1D1S Raman optical system

Mixing is characterized by measuring mole fraction of N$_2$ using a one-dimensional, one-species (1D1S) Raman scattering system. This optical system is described in Fig. 3. It comprises a continuous Coherent Verdi G18 laser producing a p-polarized laser beam at $\lambda = 532$ nm. A part of the laser beam is deviated with a Thorlabs BFS10-A beam sampler towards a Thorlabs S425C power meter to monitor the stability of the laser source. The beam is focused in the center of the combustion chamber using a convex-convex spherical lens of 750-mm focal length. The focused laser beam passes through small slits that are machined in the aluminum sidewalls of the combustion chamber and painted black to limit laser reflections. The luminosity of the laser beam, laser reflections, and the Rayleigh scattered light are filtered out by a notch optical filter (Edmund Optics $532 \pm 15$ nm OD4). The remaining scattered light is filtered around 605 nm with an OD4 Edmund Optics 86367 15 nm bandpass filter. This optical system enables to record the light scattered by N$_2$ molecules within air by Raman Stokes effect around 607 nm. This wavelength corresponds, for an excitation wavelength of 532 nm, to the only vibrational-rotational band of the dinitrogen molecule with a Raman shift equal to 2328.72 cm$^{-1}$ (Petrov 2018). Images of the Raman anti-stokes scattered signal are collected with a PCO Sensicam QE equipped with a Nikkor 105 mm f/2.8G lens. To obtain a high signal to noise ratio, pictures are recorded over a long exposure time of 40 sec. These data thus to a mean value of the nitrogen concentration probed by the laser beam. The Raman signal covers 1376 pixels horizontally over the camera sensor, providing a spatial resolution of 25 $\mathrm {\mu }$m. Each point is then spatially averaged over 20 adjacent points during the post-processing to provide a final resolution of 460 $\mathrm {\mu }$m. This post-processing choice has been made to improve the signal to noise ratio and the clarity of the figures.

## Experimental Measurements of Mixing

A parametric study is carried out to investigate the influence of the velocity ratio between the two jets $r_u = u_e/u_i$, the density ratio $r_\rho = \rho _e/\rho _i$, and the inner swirl level $S_i$ by characterizing mixing along the central axis of the burner. The experimental method based on long exposure times inherently gives access only to the mean value of the molar composition of the gas. The mixing is consequently defined here as the mean progression of the local flow composition along the vertical axis and the analysis of the results is carried out with this point of view without a priori the possibility to differentiate between segregation and mixing (Villermaux 2019). One must have however keep in mind that in the swirled flow configurations that are explored, the turbulent velocity fluctuations in the axial and azimuthal directions are extremely high, with turbulence intensity as high as $u_{y}^{rms}/\overline{u_{y}} \approx u_{\theta }^{rms}/\overline{u_{\theta }} \approx 1$ in both directions suggesting that segregation as observed in more organized flows is merely less probable. This is also confirmed by the structure of the resulting flames that are observed with the injector operated with hydrogen and air. Instantaneous OH planar laser induced fluorescence measurements carried out in Magnes et al (2024), indicate a very stable flame root above the burner outlet from laser shot to shot excluding the possibility of large vertical structure in the region that would segregate the flow.

**Table 1:** Operating conditions explored in the parametric study in Sect. 3

Annular channe/$S_{e}$	Central channel/$S_{i}$	$u_{e}$ (m/s)	$u_{i}$ (m/s)	$r_{u}$	$r_{\rho }$
Air/0.67	He/0.0	28.5	34	0.8	7.2
Air/0.67	He/0.4	28.5	34	0.8	7.2
Air/0.67	He/0.6	28.5	34	0.8	7.2
Air/0.67	He/0.9	28.5	34	0.8	7.2
Air/0.67	He/0.0	28.5	17	1.7	7.2
Air/0.67	He/0.0	28.5	34	0.8	7.2
Air/0.67	He/0.0	28.5	45	0.6	7.2
Air/0.67	He/0.6	28.5	17	1.7	7.2
Air/0.67	He/0.6	28.5	34	0.8	7.2
Air/0.67	He/0.6	28.5	45	0.6	7.2
Air/0.67	H$_{2}$/0.0	28.5	34	0.8	14.4
Air/0.67	He/0.0	28.5	34	0.8	7.2
Air/0.67	CH$_{4}$/0.0	28.5	34	0.8	1.8
Air/0.67	Ar/0.0	28.5	34	0.8	0.7
Air/0.67	H$_{2}$/0.6	28.5	34	0.8	14.4
Air/0.67	He/0.6	28.5	34	0.8	7.2
Air/0.67	CH$_{4}$/0.6	28.5	34	0.8	1.8
Air/0.67	Ar/0.6	28.5	34	0.8	0.7

The impact of the inner swirl level $S_i$ is examined first as it has been shown to control the stabilization and the structure of the flames stabilized above the HYLON injector (Marragou et al 2022, 2023b; Aniello et al 2023). The analysis then proceeds by examining the impact of the velocity ratio $r_u$ and the density ratio $r_{\rho }$ for two configurations, without inner swirl $S_i=0.0$ and with swirl $S_i=0.6$.

### Inner Swirl Level

In these experiments, the central tube is supplied with helium, which has the density closest to hydrogen but is an inert gas. The density ratio is here $r_\rho = 7.2$. The air bulk velocity is set to $u_e = 28.5$ m/s and the central bulk velocity to $u_i = 34$ m/s, corresponding to a velocity ratio $r_u = 0.8$. All these operating conditions are reported in the first part of Table 1.

**Fig. 4:**
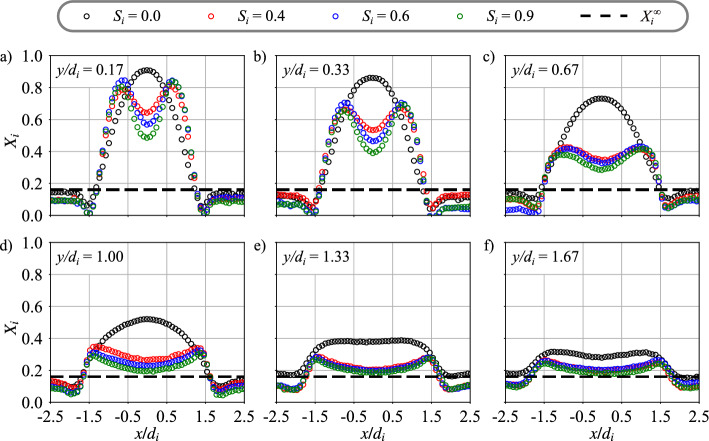
Radial profiles of helium molar fraction $X_i$ at different heights $y/d_i$ above the burner for inner swirl levels from $S_i = 0.0$ to 0.9. The air bulk velocity is $u_e = 28.5$ m/s and the central bulk velocity is $u_i = 34$ m/s, leading to a velocity ratio $r_u = 0.8$. The density ratio is $r_\rho = 7.2$. The dashed lines denotes the theoretical molar fraction of helium $X^{\infty }_{i}$ for well mixed streams

The molar fraction $X_{He}$ is deduced from the Raman scattered signal on N$_2$ molecules. Figure 4 shows the impact of the inner swirl level $S_i$ on the evolution of the molar fraction $X_i=X_{He}$ profile along the horizontal *x*-axis measured at different heights *y* above the injector. In this figure dimensions are normalized by the internal diameter $d_i$ of the central injector. The dashed horizontal line corresponds to the helium molar fraction $X_i^\infty$ of perfectly mixed jets. Figure 4 shows that mixing between the two streams occurs very rapidly, justifying the decision to focus the field of view solely on the near field of the injector outlet for distances $y/d_i<2.0$.

**Fig. 5:**
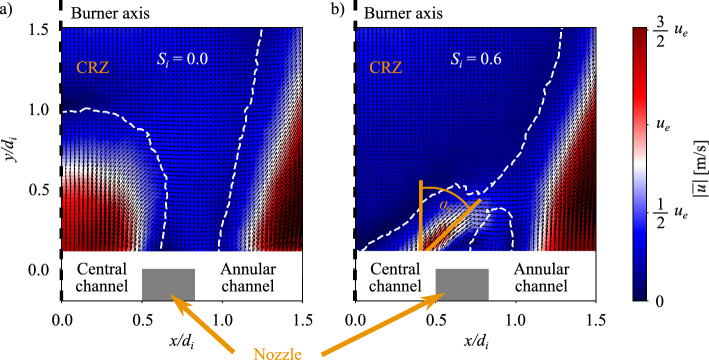
Cold flow velocity field near the injector outlet in the burner axial plane (**a**) $S_i =0.0$ and (**b**) $S_i = 0.6$. The dashed lines delineate the CRZ

Results are first examined in the absence of internal swirl $S_i=0.0$. Figure 4a shows that close to the injector outlet $y/d_i = 0.17$, the molar fraction of helium injected through the central injector is close to unity on the axis. Despite the swirl $S_e=0.67$ conferred to the external annular stream, the helium molar fraction profile is typical of a central non-swirling jet with a Gaussian shape (Galley et al 2011). It drops to zero in the wake of the central injection tube between $1.4\le |x|/d_i\le 1.6$ and re-increases again in the outer recirculation zone (ORZ) for $|x|/d_i>1.7$ to reach a value close to $X^{\infty }_{i}$, corresponding to well mixed streams. As the distance $y/d_i$ to the burner outlet increases, the molar peak on the central axis drops and the profile widens in Figs. 4b–e due to air entrainment that dilutes the central jet (Villermaux and Rehab 2000). The molar fraction profile flattens for $y/d_i = 1.33$ in Fig. 4e and becomes convex at the top for $y/d_i = 1.67$ in Fig. 4f due to the influence of the central recirculation zone (CRZ) created by the annular swirled flow (see Fig. 5a). As a conclusion, the external swirl conferred to the air stream only alters the structure of the central jet away from the injector outlet, typically at distances larger than the internal diameter $y\sim d_i$.

When swirl is imparted to the central flow, i.e. $S_i\ne 0.0$, radically different profiles are observed in Fig. 4 and indicate much faster mixing. Even close to the injector outlet at $y/d_i = 0.17$, the profile takes a M-shape in Fig. 4a for the three swirl levels tested $S_i=0.4$, 0.6, and 0.9. In the external shear layer of the annular jet and inside the ORZ, they are similar to that observed without inner swirl $S_i=0.0$. The main modification is close to the center. In the central region of the flow, two symmetric peaks are visible, and a drop of the molar fraction of helium is measured around the centerline. The symmetric peaks are due to the radial expansion of the central jet when swirl is imparted to the central flow. The decrease of the molar fraction of helium around the centerline is due to the penetration of the CRZ in the upstream direction up to the central injector outlet section, as confirmed by particle image velocimetry measurements shown in Fig. 5b. For $y/d_i = 0.17$, peak values are close to the maximum reached along the centerline without inner swirl. Increasing the distance *y* to the injector outlet, the difference between the peak values and the molar fraction reached on the centerline decreases in Figs. 4b–c. When swirl $S_i>0.0$ is imparted to the central jet, the maximum value of the molar fraction of helium decreases much more rapidly than in the unswirled case $S_i=0.0$. For $y/d_i = 0.67$, the maximum molar fraction reached for the swirled cases is half of the value measured without inner swirl in Fig. 4c. However, the molar fraction profiles are similar for all the swirl levels $S_i= 0.4$, 0.6, and 0.9 tested.

These experiments confirm that conferring a swirl motion to the central stream drastically improves the mixing of the coaxial jets. The impact of $S_i>0.0$ is particularly notable close to the injector outlet. The molar fraction of helium is only two times the perfectly mixed limit $X_i^\infty =0.16$ in Fig. 4c at $y/d_i=0.67$ and well mixed conditions are almost reached in Fig. 4e at $y/d_i=1.33$, while perfectly mixed conditions are still not achieved for the non-swirling jet $S_i=0.0$ in Fig. 4f at $y/d_i=1.67$.

### Velocity Ratio

Experiments are further conducted with helium in the central channel and air in the annular channel. The influence of the velocity ratio $r_u = u_e/u_i$ is now investigated by testing three values of $r_u$. This is done for two configurations of the central injector, without swirl $S_i=0.0$ and with a swirl level $S_i=0.6$. The operating conditions explored in this subsection are reported in the second part of Table 1. The objective is to compare the impact of $r_u$ on mixing for non-swirling and swirling inner jets.

**Fig. 6:**
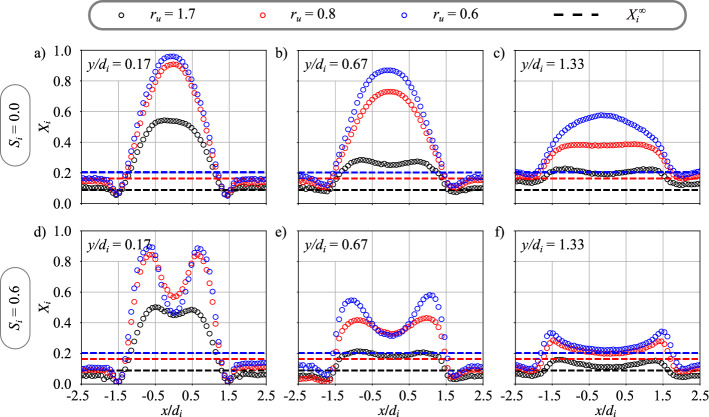
Radial profiles of helium molar fraction $X_i$ at different heights $y/d_i$ above the burner for three central injection velocities $u_i = 17$ m/s ($r_u = 1.7$), 34 m/s ($r_u=0.8$), and 45 m/s ($r_u=0.6$) (see second part of Table 1). The density ratio is $r_\rho = 7.2$. (**a**) to (**c**) $S_i = 0.0$. (**d**) to (**f**) $S_i = 0.6$. The dashed lines are the theoretical molar fractions of helium $X^{\infty }_{i}$ for well mixed streams

Figure 6 shows the molar fraction radial profiles of helium $X_i$ with a velocity ratio varied from $r_u = 0.6$ to 1.7 for axial positions $y/d_i=0.17$, 0.67, and 1.33. For readability reasons, results are only presented up to $y/d_i = 1.33$ where the mixing between the central and the annular jet is not completed, but the trends are clear. The annular air bulk velocity is fixed to $u_e = 28.5$ m/s and the central injection velocity is set successively to $u_i=17$, 34, and 45 m/s to modify the velocity ratio $r_u=1.7$, 0.8, and 0.6 respectively.

In the top row of Figs. 6a to c, results are presented for central injection without swirl $S_i = 0.0$. Mixing progresses monotonically with the distance *y* from the injector outlet along the centerline. It also progresses faster when the velocity ratio $r_u$ increases, i.e. when the central injection velocity is reduced in accordance with previous studies (Chriss and Paulk 1972; Rehab et al 1997; Villermaux and Rehab 2000; Schumaker and Driscoll 2012). For the case without swirl motion conferred to the central flow, these measurements confirm than, instead the annular stream is swirled, the mixing between the two coaxial jets behaves qualitatively as for a configuration where the annular stream is unswirled, configuration even well assessed in the scientific literature.

Results at the bottom row of Figs. 6d to f are obtained for a swirled central injection with $S_i = 0.6$. These plots confirm the observations made in Fig. 4 with molar fraction profiles that differ radically from the $S_i=0.0$ cases and take a M-shape. The two peaks of molar fractions on the side of the profiles also decrease when the velocity ratio $r_u$ increases. Modifying the velocity ratio alters the molar fraction profiles for $S_i=0.6$ in a very similar way as for $S_i=0.0$. Additional experiments, not shown here, were carried out by fixing the central injection velocity $u_i$ and varying $u_e$ leading to same observations. These results provide an important information on the behavior of the mixing of dual swirl coaxial jets. It is shown that, instead the near-field flow structure of the central jet is substantially altered by the swirl motion of this later, the velocity ratio $r_{u}$ previously identified as a driving parameter for the mixing of coaxial unswirled jets is still a primary parameter and the mixing is altered in the same way when this parameter is varied.

**Fig. 7:**
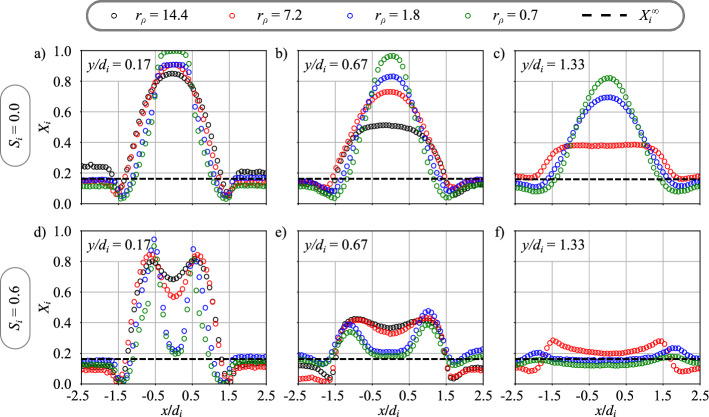
Radial profiles of molar fraction $X_i$ at different heights $y/d_i$ above the burner for three gases injected through the central tube: hydrogen ($r_\rho = 14.4$), helium ($r_\rho = 7.2$), methane ($r_\rho = 1.8$), and argon ($r_\rho = 0.7$). The central injection velocity is $u_i = 34$ m/s and the annular bulk velocity is $u_e = 28.5$ m/s leading to a velocity ratio $r_u = 0.8$. (**a**) to (**c**) $S_i = 0.0$. (**d**) to (**f**) $S_i = 0.6$. The dashed lines are the theoretical molar fractions $X^{\infty }_{i}$ for well mixed streams

### Density Ratio

The influence of the density ratio $r_\rho = \rho _e/\rho _i$ on mixing is now investigated for an annular air bulk velocity $u_e = 28.5$ m/s and a central injection velocity $u_i = 34$ m/s, corresponding to a velocity ratio $r_u = 0.8$. The corresponding operating conditions are indicated in the third part of Table 1.

Figure 7 shows the evolution of the profiles of the molar fraction $X_i$ of the gas injected in the central channel at three heights above the burner when the density ratio between the annular and the central flow is varied from $r_\rho = 0.7$ to 14.4. To achieve this variation, the gas injected through the central injector is switched between hydrogen ($r_\rho = 14.4$), helium ($r_\rho = 7.2$), methane ($r_\rho = 1.8$), and argon ($r_\rho = 0.7$). Flow conditions are reported in the second part of Table 1. The hydrogen case (black symbols, $r_\rho = 14.4$) is not available for the height $y/d_i = 1.33$ in Figs. 7c and f.

Cases without inner swirl $S_i = 0.0$ are shown in the top row of Figs. 7a to c. These figures show that the density ratio $r_\rho$ has a strong impact on the mixing between the two flows. For the same velocity ratio $r_u = 0.8$, decreasing the density ratio $r_\rho$ by increasing the density of the gas injected in the center leads to a decrease of the mixing rate corroborating previous studies (Chriss and Paulk 1972; Rehab et al 1997; Villermaux and Rehab 2000). This behavior results from the increase of the momentum of the central jet with the density of the inner gas, leading to a higher penetration of the jet into the CRZ and consequently a decrease of the mixing rate.

The impact of the inner swirl $S_i = 0.6$ is shown at the bottom row of Figs. 7d to f. Close to the injector outlet at $y/d_i=0.17$, the distribution of the molar fraction on the burner axis in Fig. 7d is opposite to the one found without inner swirl in Fig. 7a when the density ratio increases from $r_\rho =0.7$ to 14.4. The heaviest gas, argon, which protrudes the further inside the CRZ in absence of inner swirl, penetrates the least inside the flow when swirl is conferred to the central stream. This behavior along the burner axis is again caused by a change of the momentum of the central jet. Except close to the injector along the burner axis, modifying the density ratio only weakly alters the peaks in the external shear layers in Figs. 7d to f. It is remarkable that the flow approaches well mixed conditions in Fig. 7f at $y/d_i=1.33$ for all cases explored. However in Figs. 7d to f, for $S_i = 0.6$, the position of the peaks of molar fraction does not depend on the density ratio in the near field of the injector outlet for $y/d_i < 1.0$. In Figs. 7a to c, for $S_i = 0.0$, the width of the peaks increases slightly with the density ratio. However as for the case with swirl imparted to the central stream, the deflection angle of the jet remains roughly constant when the density ratio is varied. These observations are corroborated by PIV measurements from Marragou (2023) with different gases (air and helium) injected in the central channel.

These figures confirm the weaker influence of the density ratio on the mixing progress compared to changes of the velocity ratio. The observations are used as a starting point for the derivation of a model for mixing.

## Analysis of the Mixing Progress

The previous results are analyzed with the help of the mixing progress *C* defined by Eq. (1).

In the core of the central jet close to the injector outlet, mixing has not started, in which case max$\left( X_{i} \right) =1$ and $C=0$. Far away from the injector outlet, the two streams are well mixed, implying that max$\left( X_i \right) = X^{\infty }_{i}$ and mixing is complete with $C=1$. Intermediate values $0<C<1$ indicate the progress of mixing as a function of the distance *y* to the injector outlet.

In this model, the maximum value of the molar fraction $X_i$ of the gas injected through the central channel is chosen to describe the progress of the mixing. Another possibility would have been to determine the mean value of the mixture fraction by averaging the profile over the central jet width as done in Villermaux and Rehab (2000) or by considering only the value on the centerline (Papadopoulos and Pitts 1998). The first approach is more difficult to achieve here due to the presence of a CRZ, in which case it is difficult to delineate with precision the boundaries of the central jet. However, tests have been made on a few selected cases and the two methods yield very similar results. The second approach is not appropriate for the case of swirled central jets, which expand radially. The adopted approach is however equivalent to the one used by Papadopoulos and Pitts (1998) where the value of the concentration on the centerline corresponds roughly to the maximum value for unswirled jets. Moreover, according to previous studies with unsteady concentration measurements as in Villermaux and Rehab (2000), the maximum value of the concentration profiles corresponds to the core of the jets where concentration fluctuations are minimal.

The influence of the inner swirl level $S_i$, velocity ratio $r_u$, and density ratio $r_\rho$ are investigated by examining how *C* progresses with the distance $y/d_i$ above the injector. Experiments are repeated for two injection strategies by keeping the velocity ratio $r_u = u_e/u_i$ constant in a first series and then the momentum ratio $J = \rho _e u_e^2/\rho _i u_i^2 = r_\rho r_u^2$ in a second series.

**Table 2:** Velocity ratios $r_{u} = u_{e}/u_{i}$ at constant momentum ratio $J = 10.1$ for different gas injected through the central tube

Annular channel	Central channel	*J*	$r_\rho$	$u_{e}$ (m/s)	$u_{i}$ (m/s)	$r_{u}$
Air	Hydrogen	10.1	14.4	28.5	34.0	0.8
Air	Methane	10.1	1.8	28.5	12.1	2.4
Air	Argon	10.1	0.7	28.5	7.6	3.8

**Fig. 8:**
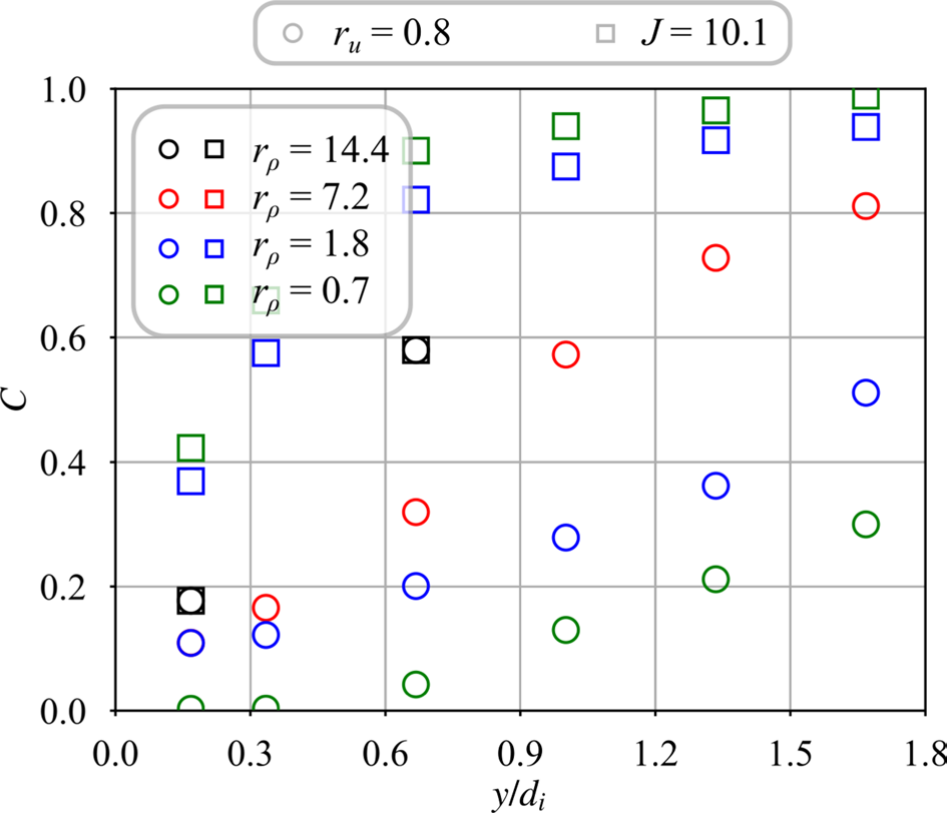
Mixing progress *C* for unswirled central jets $S_{i} = 0.0$ and different density ratios $r_{\rho } = 0.7$ to 14.4. For the cases $J = 10.1$, the velocity ratio is varied from $r_{u} = 0.8$ to 3.8

In the two series of experiments, the air velocity is fixed to $u_e=28.5$ m/s. For experiments at the fixed momentum ratio $J=10.1$, the central injection velocity $u_i$ is adapted depending on the density $\rho _i$ of the gas flowing inside the central tube according to values reported in Table 2. When the velocity ratio is kept constant to $r_u=0.8$, only the inner gas density $\rho _i$ is varied by injecting hydrogen, helium, methane, and argon through the central channel.

Figure 8 shows how mixing progresses with the distance $y/d_i$ above the burner when there is no swirl imparted to the central jet, $S_i = 0.0$. Square symbols show results when *J* is kept constant and circles denote those obtained at constant velocity ratio $r_u$. This figure indicates that the mixing progress depends both on velocity ratio $r_u$ and density ratio $r_\rho$, but also on the injection strategy.

When the velocity ratio is fixed to $r_u = 0.8$ (circle symbols) and the density ratio is varied, mixing progresses faster in Fig. 8 for light gases injected through the central tube. Far away from the injector outlet at $y/d_i = 1.67$, mixing progresses two time faster for $r_\rho = 7.2$ (helium) than for $r_\rho = 0.7$ (argon). Moreover for argon ($r_\rho = 0.7$) and methane ($r_\rho = 1.8$), mixing begins further downstream above the injector outlet at $y/d_i \approx 0.3$, than with the use of helium ($r_\rho = 7.2$) or hydrogen ($r_\rho = 14.4$) through the central tube.

When the momentum ratio is fixed at $J = r_\rho r_u^2= 10.1$ (square symbols) and the density ratio $r_\rho$ is varied, the velocity ratio $r_u$ increases rapidly due to the drop of the density ratio (see Table 2) and mixing progresses faster. Figure 8 shows that the dispersion of the data for $J = 10.1$ is less than for injection at constant velocity ratio $r_u = 0.8$ when there is no central swirl $S_i = 0.0$. This observation corroborates previous studies (Chriss and Paulk 1972; Rehab et al 1997; Villermaux and Rehab 2000) identifying the momentum ratio *J* as the primary driving parameter for the mixing of coaxial jets in the absence of swirl. However, the square symbols do not collapse on the same curve in Fig. 8 as in the case of non swirling jets. The swirl motion conferred to the annular flow with $S_e=0.67$ leads to the establishment of a CRZ that alters mixing.

**Fig. 9:**
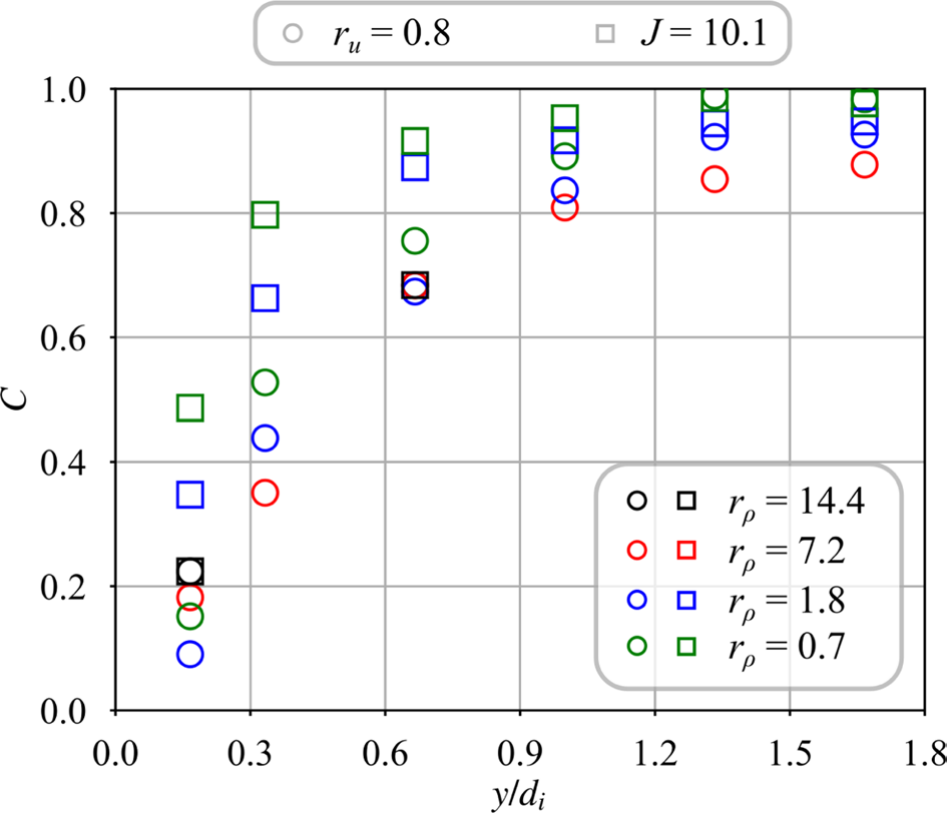
Mixing progress variable for a swirled central jet $S_{i} = 0.6$ with the density ratio varying from $r_\rho = 0.7$ to 14.4. For the cases $J = 10.1$, the velocity ratio is varied from $r_{u} = 0.8$ to 3.8 (see Table 2)

Figure 9 shows how mixing is altered with a swirl motion $S_i = 0.6$ impregnated to the central flow. Results are plotted for the same injection conditions but data are less dispersed than for the unswirled case $S_i = 0.0$ in Fig. 8. Dispersion of the data is a bit less for the injection strategy $r_u = 0.8$ than for $J = 10.1$. The differences are however small and confirm the previous observations made in Fig. 7. Modifying the density ratio $r_\rho$ has minimal effect on the mixing when the central jet is swirled. In this scenario, the velocity ratio $r_u$ appears to be the main parameter controlling the mixing between the two swirling jets.

**Fig. 10:**
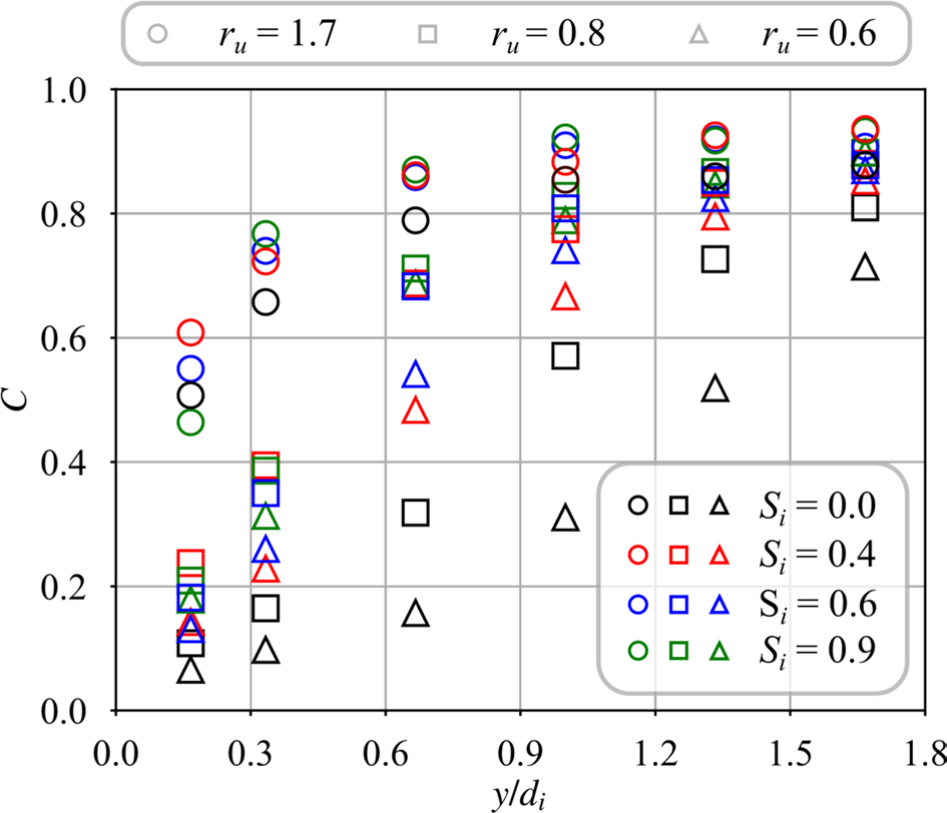
Mixing progress variable for helium ($r_\rho = 7.2$) with a variable swirl level of the central jet from $S_{i} = 0.0$ to $S_{i} = 0.9$. The velocity ratio is varied from $r_{u} = 0.6$ to $r_{u} = 1.7$

The density ratio is now set to $r_\rho = 7.2$ with helium injected in the central tube and the velocity ratio $r_u$ is varied for different inner swirl levels $S_i$. Results are presented in Fig. 10. Again, progress of mixing appears to be mainly correlated to the velocity ratio $r_u$, except for $S_i = 0.0$. Independently of the inner swirl level $0.4\le S_i \le 0.9$, mixing progresses faster for high values of velocity ratio $r_u$.

## Modeling the Mixing Progress

**Fig. 11:**
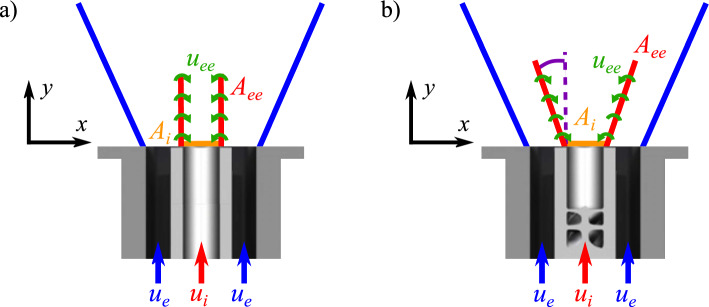
Schematics illustrating the modeling approach adopted for the scaling of the mixing progress variable *C*. (**a**) Original model from Villermaux and Rehab (2000) valid for $S_{i} = 0.0$ and $S_e = 0.0$. (**b**) Adapted model for the dual swirl injection with $S_{i} \ge 0.0$ and $S_{e} > 0.0$

The model proposed by Villermaux and Rehab (2000) for the mixing of non-swirling co-axial jets with thin lips is revisited and tailored to suit the cases of swirled flows. A schematic of the main geometrical and flow parameters of the original model is shown in Fig. 11a. The geometry is considered to be axi-symmetric with a straight central jet exhausting from the central channel of cross section area $A_i=\pi d_i^2$ discarding jet divergence, $\alpha _i = 0^{\circ }$. The model relies on a lateral surface exchange denoted $A_{ee}$ between the central and annular streams depicted by the red line. It considers that the central jet is diluted along the vertical *y*-axis by entrainment of gas from the annular co-flow with an entrainment velocity $u_{ee}$ along the cylindrical surface of area $A_{ee}$. From a physical point of view, this entrainment velocity represents the mean rate of mass exchange between the two jets due to large vortical structures. The entrainment velocity $u_{ee}$ is assumed to be proportional to the annular bulk velocity $u_e$ of the external annular jet, $u_{ee} \propto u_e$, and the velocity ratio is assumed to be much greater than unity, $r_u>> 1$.

With these different hypotheses and from the definition of the molar fraction $X_i$ of the gas injected through the central tube at height *y* above the burner, one may write (Villermaux and Rehab 2000):

2 $$\begin{aligned} X_i = \frac{\dot{n}_i}{\dot{n}_i + \dot{n}_e} \sim \frac{u_i A_i}{u_i A_i + u_{ee} A_{ee}} \end{aligned}$$

where $\dot{n}_i$ and $\dot{n}_e$ are the molar flowrates in the inner and annular channels respectively.

In the case of a straight cylinder and $r_u>> 1$, Eq. (2) leads to:

3 $$\begin{aligned} X_i \sim \left( \left( \frac{y}{d_i} \right) r_u\right) ^{-1} \end{aligned}$$

The same modeling approach is retained here and adapted to the case of swirled flows. The assumption $r_u>> 1$ is relaxed, but the exchange surface area $A_{ee}$ corresponding to the interface between the central and the annular jets is modeled as an inverted truncated cone with an angle $\alpha _i$ as shown in Fig. 11b:

4 $$\begin{aligned} A_{ee} = \pi \left( y \tan \left( \alpha _i \right) + d_i \right) \frac{y}{\cos \left( \alpha _i \right) } \end{aligned}$$

The main difference with the original model is to take into account the new topology of the central swirling jet featuring an angle $\alpha _i$ that increases when the internal swirl number $S_i$ increases. When $\alpha _i=0$, one retrieves the surface area $A_{ee}=\pi d_i y$ considered by Villermaux and Rehab (2000) for straight non-swirling jets.

Substitution of Eq. (4) into Eq. (2) leads to the following modified expression for $X_i$:

5 $$\begin{aligned} X_i \sim \left[ 1 + 4 \delta \left( \frac{y}{d_i} \tan \left( \alpha _i \right) + 1 \right) \left( \frac{y}{d_i} \right) \frac{r_u}{\cos \left( \alpha _i \right) } \right] ^{-1} = \xi \end{aligned}$$

where the parameter $\delta$ corresponds to the ratio $\delta = u_{ee}/u_e$ between the entrainment velocity $u_{ee}$ and the bulk air velocity $u_{e}$ in the annular channel. The quantity in the right hand side in Eq. (5) is denoted $\xi$. The theoretical mixing progress variable *C* calculated with $\xi$ is denoted $\eta$:

6 $$\begin{aligned} C \sim \left( 1 - \xi \right)/\left( 1 - X^{\infty }_{i} \right) = \eta \end{aligned}$$

This relation is equivalent to Eq. (3) from Villermaux and Rehab (2000) when $r_u>> 1$ and $\alpha _i = 0^{\circ }$. The parameter $\delta$ in Eq. (5) needs to be determined here because the simplification $r_u>> 1$ cannot be used for the range of flow conditions explored in this study. The angle $\alpha _i$ and velocity ratio $\delta$ are deduced from measurements as explained below.

PIV measurements from Marragou et al (2023a) conducted with the same injector with helium and air revealed that the helium central jet expands radially directly at the outlet of the injector even for cases without inner swirl $S_i = 0.0$. Two flow velocity fields are exemplified in Fig. 5, with the angle of the central jet $\alpha _i$ schematically represented in Fig 5b. The angle of this jet differs from $\alpha _i=0^o$ as reported in Table 3. The following hypotheses are made to simplify the problem: (i) When $Si=0.0$, the angle $\alpha _i$ is considered to be independent of the velocity ratio $r_u$ and (ii) When $S_i\ne 0$, $\alpha _i$ is assumed to only depend on the inner swirl level $S_i$ and the velocity ratio $r_u$. When $S_i=0.0$, the angle $\alpha _i$ is small (Table 3). It has been checked that the results are barely modified when the angle varies between $5^o \le \alpha _i \le 12^o$, indicating that hypothesis (i) Is reasonable. Hypothesis (ii) Is more difficult to justify. It has however been checked that the jet angle $\alpha _i$ does not depend on the density ratio $r_\rho$ (see Fig. 7).

The entrainment velocity ratio $\delta$ is more difficult to estimate and is fitted in this study with mixing measurements made for the hydrogen case with $S_i = 0.6$, $u_e = 28.5$ m/s, and $u_i = 34$ m/s, i.e. $r_u=0.8$ and $r_\rho =14.4$, in order to match the theoretical expression $\eta$ with the measured progress variable *C* deduced from Raman scattering measurements. The best match is obtained for $\delta = 0.25$. This value is kept constant in Eq. (5) for all other operating conditions explored in this study. This is admittedly a rough approximation, but it will be shown to reasonably well reproduce the observations made.

**Table 3:** Angles $\alpha _i$ of the central jet with respect to the vertical *y*-axis deduced from PIV measurements from Marragou et al (2023a) for helium and air $r_\rho =7.2$. These values are used in Figs. 12, 13 and 14

$S_{i}$	$u_{i}$ (m/s)	$r_{u}$	$\alpha _{i} (deg)$
0.0	All	All	8
0.4	34	0.8	32
0.6	17	1.7	64
0.6	34	0.8	41
0.6	45	0.6	50
0.9	34	0.8	48

**Fig. 12:**
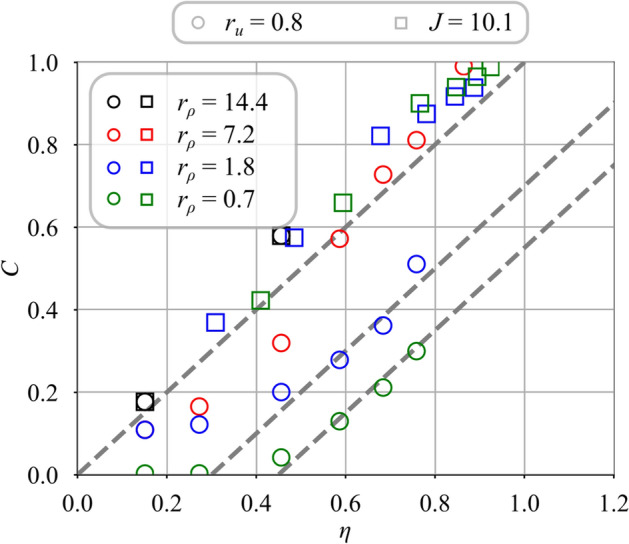
Comparison between measured *C* and modeled $\eta$ mixing progress without swirl conferred to the central stream $S_i = 0.0$. For the cases with $J = 10.1$, the velocity ratio is varied from $r_{u} = 0.8$ to 3.8. Data correspond to that presented in Fig. 8

In the following comparisons, the air bulk velocity $u_e$ is kept constant and equal to $u_e = 28.5$ m/s. Data gathered without swirl $S_i = 0.0$ in the central injector from Fig. 8 are considered first. The angle $\alpha _i$ used for the determination of the mixing scaling variable $\eta$ is $\alpha _i = 8^{\circ }$ for all operating conditions. The mixing progress *C* is plotted in Fig. 12 when the velocity ratio is varied from $r_u = 0.8$ to $r_u = 3.8$ and the density ratio from $r_{\rho } = 0.7$ to $r_{\rho } = 14.4$ for the two injection strategies. Figure 12 shows that measurements of *C* scale linearly with the theoretical estimate $\eta$ with the same unity slope, independently of the strategy selected to inject the gas, i.e. $J=10.1$ or $r_u=0.8$. The reference case is hydrogen, with $r_u=0.8$ and $r_\rho =14.4$ for which *C* has been matched with $\eta$. Results for the light gases, hydrogen ($r_\rho =14.4$) and helium ($r_\rho =7.2$), and low central injection velocities, $r_u>2$ collapse on the same line crossing zero. Data for methane ($r_\rho =1.8$) and argon ($r_\rho =0.7$) injected at $r_u=0.8$ follow a straight line of unity slope, but they intercept the $\eta$-axis at a value shifted to the right, indicating a delayed onset of mixing. This late onset is attributed to the larger momentum of the central jet when the gas density $\rho _i$ is increased and the injection velocity $u_i$ is kept constant. In these cases, the higher momentum of the central jet for the heaviest gases, i.e. methane and argon, leads to a wider penetration of the central jet inside the CRZ as confirmed by the PIV measurements from Marragou (2023). The inner potential core depicted in Fig. 1 and shown in Fig. 5a thus extends further downstream into the flow for these cases, with a delayed onset of mixing with respect to cases with a narrower inner potential core for which mixing already starts very close to the injector outlet.

Results in Fig. 12 validate the modeling approach for cases without inner swirl, $S_i=0.0$, extending the validity of the model from Villermaux and Rehab (2000) to the configurations of coaxial jets with swirl conferred to the annular air channel, i.e. configurations with vortex breakdown and a CRZ that settles at a distance of one diameter away from the injector outlet.

**Fig. 13:**
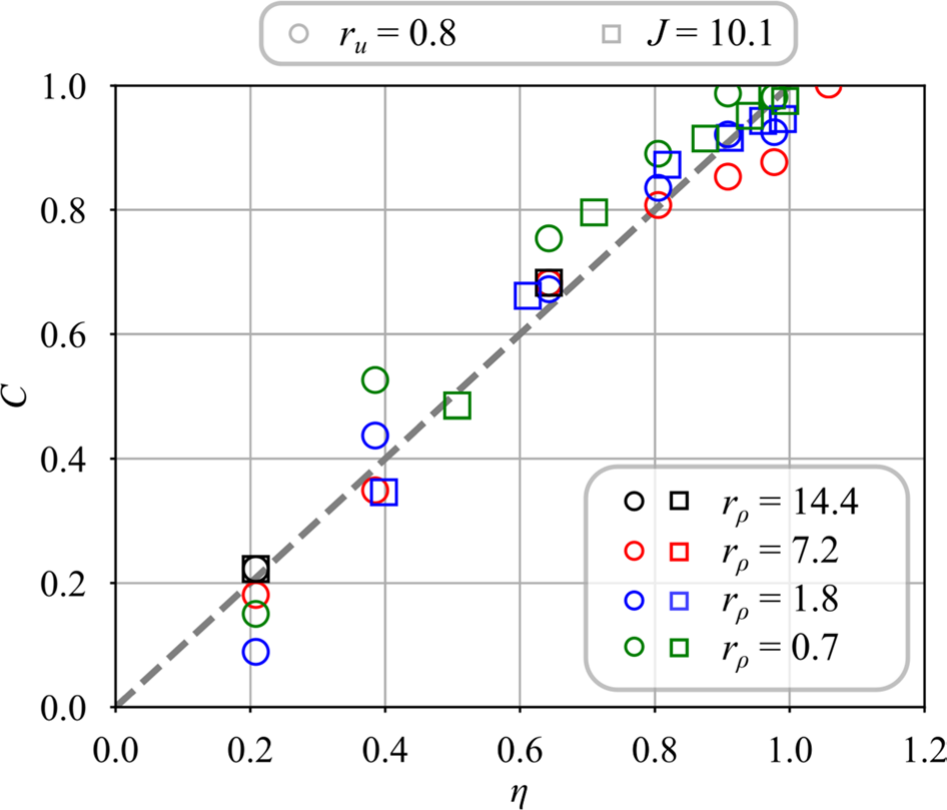
Comparison between measured *C* and modeled $\eta$ mixing progress with swirl conferred to the central stream $S_i = 0.6$. For the cases with $J = 10.1$, the velocity ratio is varied from $r_{u} = 0.8$ to 3.8. The data correspond to that presented in Fig. 9

The swirl level in the central channel is now fixed to $S_i = 0.6$. Data from Fig. 9 are rescaled with respect to $\eta$ which is estimated with the same value of the entrainment velocity ratio $\delta =0.25$ and measured jet angles $\alpha _i$ taken from Table 3. Results presented in Fig. 13 show that the measured mixing progress *C* scales well with the estimate $\eta$ for all data and roughly falls on a single line of unity slope crossing zero. In all cases, the jets mix directly at the outlet of the injector due to the penetration of the CRZ inside the central injector as shown in Fig. 5b. This figure confirms that the model in Eq. (5) is able to reproduce mixing even when an inner swirl, $S_i\ne 0.0$, is conferred to the central jet.

**Fig. 14:**
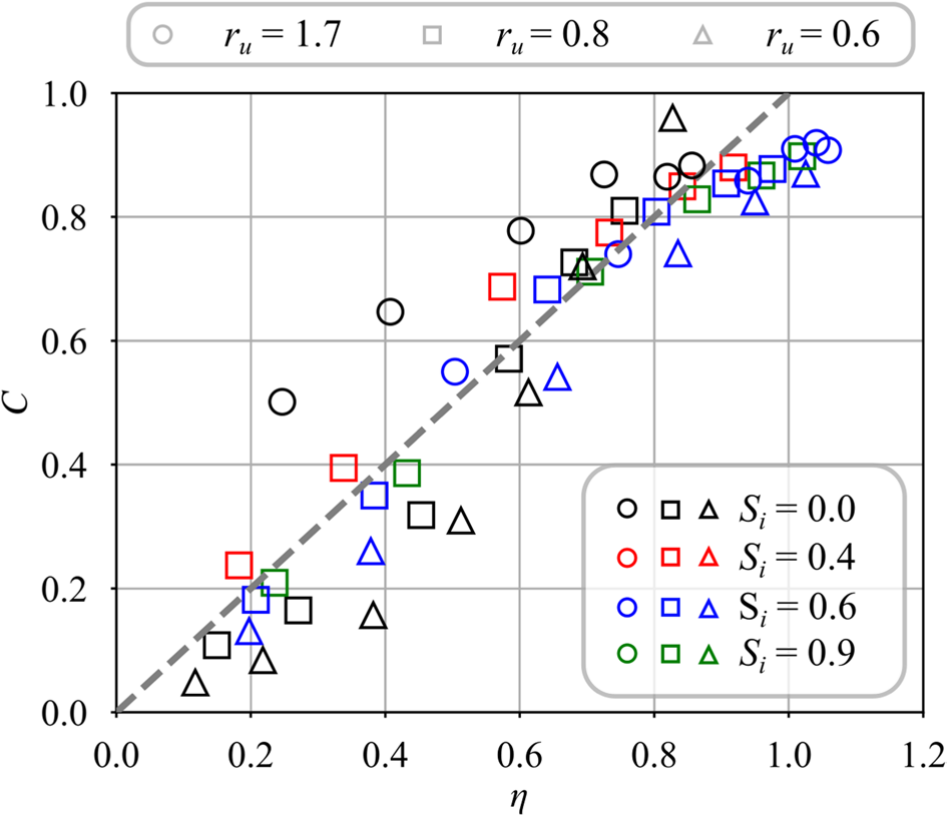
Mixing progress variable for helium injected in the central channel with a swirl level varied from $S_i = 0.0$ to 0.9. The velocity ratio is varied from $r_u = 0.6$ to 1.7. Results are plotted against the mixing scaling variable $\eta$. The data correspond to that presented in Fig. 10

Predictions of the model are now compared to a larger set of measurements with an inner swirl level varying from $S_i = 0.0$ to 0.9 and a velocity ratio from $r_u = 0.6$ to 1.7. The gas injected through the central channel is helium, corresponding to a fixed value of the density ratio $r_\rho = 7.2$. Data from Fig. 10 are rescaled as a function of $\eta$ in Fig. 14. The measured mixing progress *C* falls roughly on the same line with a unity slope crossing the origin when it is plotted as a function of $\eta$. However, relatively large deviations are also observed for cases without inner swirl $S_i = 0.0$. They are attributed to the perturbation of the central jet induced by the CRZ produced by the swirling external jet, acting as a counter-flow on the expanding central jet (see Fig. 5a). The largest deviation with respect to $\eta$ is observed for the largest velocity ratio $r_u = 1.7$, corresponding to the smallest central injection velocity $u_i$. PIV measurements from Marragou (2023) indicate that the momentum ratio between the central jet and the CRZ along the centerline of the injector is in this case less than unity meaning that the jet is weaker than the recirculating flow, i.e. the central jet is strongly disturbed by the recirculating flow inside the CRZ. This mechanism may explain the observed differences. The assumption of a constant value of the entrainment velocity ratio $\delta$ can also play a role in these discrepancies. Further investigation by, for example, varying the external swirl level $S_e$ would be necessary to clarify this issue. However, Fig. 14 confirms that $\eta$ describes reasonably well the mixing progress for the range of operating points explored in this study to further interpret mixing between swirled jets.

## Conclusion

Mixing between dual swirled central and external jets of the HYLON injector has been investigated in cold flow conditions in order to better understand how the inner swirl level $S_i$, the velocity ratio $r_u$, and the density ratio $r_\rho$ affect the mixing between the central and annular flows. The results can be summarized as follows:

- The inner swirl motion $S_i > 0.0$ increases substantially the mixing rate between the central and the annular streams, particularly in the first millimeters above the central injector outlet.
- Increasing the swirl level above $S_i \ge 0.6$ only marginally enhances mixing compared to a moderate swirl level, for example $S_i = 0.4$.
- Increasing the velocity ratio $r_u$ improves mixing for all inner swirl levels investigated.
- Increasing the density ratio $r_\rho$ enhances mixing when the central jet is not swirled $S_i = 0.0$ but has a negligible impact on the mixing rate when swirl is conferred to the central jet $S_i \ne 0.0$.
- A model inspired from Villermaux and Rehab (2000) has been developed and tailored to include the impact of the inner swirl level $S_i$, the velocity ratio $r_u$, and the gas density ratio $r_\rho$ leading to a theoretical expression for the mixing progress $\eta$.
- The mixing progress $\eta$ depends on the expansion angle $\alpha _i$ of the central jet and the ratio $\delta$ between the entrainment velocity $u_{ee}$ and the external air velocity $u_e$. The angle $\alpha _i$ was determined in this study from PIV measurements and $\delta$ was fitted with measurements made with hydrogen for a single flow operating conditions.
- The model $\eta$ has been shown to reproduce well the mixing progress *C* with data collapsing reasonably on a single line of unity slope crossing the origin, provided that mixing between the two jets starts at the burner outlet. The largest differences are found for the unswirled cases $S_i=0.0$. It has been hypothesized that the disturbance caused by the CRZ acting as a counter-flow on the central jet partly is at the origin of these discrepancies.

The analysis conducted in this work improves the understanding of the mixing of coaxial jets when swirl is conferred to the central stream. The results may be used to guide the design of new generations of hydrogen coaxial injectors.

However, one needs to recall some of the main limitations of the observations and conclusions. Only the mean value of the molar fraction of the species injected in the central stream has been considered due to technical limitations of the Raman scattering measurement chain while unsteady structures may also play a role in the mixing process of sheared flows. This approach is considered in this study as a good macroscopic representation of the mixing between the two streams. Their influence is herein only taken into account as an average effect through a mean entrainment velocity. The annular swirl level and the injector diameters have also been kept constant. An extension of the study for different values of the annular swirl level and for injectors with different diameters would enable a consolidated validation of the model. Finally, direct measurements of the entrainment velocity $u_{ee}$ would be useful in future studies.

## Data Availability

The data that support the fndings of this study are available from the corresponding author upon reasonable request.
